# Reduced CH25H expression defines a high membrane fluidity, trogocytosis active state in colon cancer stem cells

**DOI:** 10.3389/fcell.2026.1834358

**Published:** 2026-07-09

**Authors:** Minho Jang, Yoonseo Park, Jeonghyun Kim, Soyeon Park, So-Hyeon Park, Taekeun Kim, Hojin Lee, Jungmin Choi, Jae Hun Shin

**Affiliations:** 1 Department of Integrative Biotechnology, Yonsei University, Incheon, Republic of Korea; 2 Department of Integrated Science and Engineering Division, Yonsei University, Incheon, Republic of Korea; 3 The Interdisciplinary Graduate Program in Integrative Biotechnology, Yonsei University, Incheon, Republic of Korea; 4 Department of Biomedical Sciences, Korea University College of Medicine, Seoul, Republic of Korea

**Keywords:** cancer stem cells, CH25H, membrane fluidity, spheroid, trogocytosis

## Abstract

**Introduction:**

Trogocytosis enables direct membrane exchange between contacting cells and has recently been implicated in tumor immune evasion. However, the cellular states and molecular mechanisms that regulate cancer cell trogocytosis remain largely unknown. Here, we investigated whether trogocytosis differs between cancer stem cells (CSCs) and non-stem cancer cells (non-CSCs) in colorectal cancer.

**Methods:**

Three-dimensional spheroid cultures of Caco-2, HCT116, and HT-29 colorectal cancer cells were used to enrich CSCs. Trogocytosis was quantified by co-culture with membrane-labeled Jurkat T cells via flow cytometry. Membrane fluidity, gene expression, STAT1 phosphorylation, and publicly available human colorectal cancer single-cell RNA-seq datasets were analyzed to investigate the underlying mechanisms.

**Results:**

3D spheroid culture enriched CSC populations and markedly increased trogocytic membrane transfer from Jurkat T cells to tumor cells. Within spheroids, CSCs acquired significantly higher levels of T cell-derived membrane proteins and membrane components than non-CSCs, indicating preferential enhancement of trogocytosis in CSCs. Membrane order analysis revealed reduced membrane order and increased membrane fluidity in CSCs. Quantitative PCR analysis demonstrated reduced expression of cholesterol 25-hydroxylase (CH25H) and cholesterol efflux-related genes in CSCs Because CH25H is an interferon-responsive gene downstream of STAT1 signaling, we next examined STAT1 activity. CSCs exhibited reduced STAT1 phosphorylation, which was associated with enhanced trogocytic membrane acquisition. Furthermore, activation of STAT1 signaling by IFN-γ reduced the CSC population and suppressed trogocytosis. Analysis of human colorectal cancer single-cell RNA-sequencing datasets further supported an association between reduced STAT1 signaling and decreased CH25H expression.

**Discussion:**

These findings identify a CH25H-low CSC state characterized by increased membrane fluidity and enhanced trogocytosis. Reduced STAT1-CH25H signaling may promote membrane biophysical remodeling that facilitates trogocytosis in colon cancer stem cells and contributes to tumor progression and immune evasion.

## Introduction

Trogocytosis is a dynamic and contact-dependent cellular process in which a cell acquires plasma membrane fragments, including functional membrane proteins, from another cell during direct contact (Joly and Hudrisier, 2003). Unlike phagocytosis, the transferred membrane proteins retain their surface localization and functional activity in recipient cells. In the tumor microenvironment, cancer cells can acquire immune-regulatory molecules such as CTLA-4 and TIM-3 via trogocytosis, thereby contributing to immune evasion (Shin et al., 2021a). In addition, recent studies have shown that tumor cells impair chimeric antigen receptor (CAR)-T cell function by acquiring CAR molecules through trogocytosis (Lu et al., 2022; Schoutrop et al., 2022). While these studies demonstrate that cancer cells are capable of trogocytosis, most prior research has primarily focused on immune cell-mediated trogocytosis. Therefore, whether distinct tumor cell subtypes differ in their intrinsic ability to perform trogocytosis remains unknown.

In our previous study, we demonstrated that carcinogenesis promotes trogocytosis activity (Shin et al., 2021b). Using genetically defined murine colon cancer organoids, we showed that organoids carrying mutations in *Apc, Kras, Tp53,* and *Smad4* genes exhibited approximately a two-fold increase in trogocytic activity compared with organoids carrying *Apc* mutation alone. Given that the quadruple mutant organoids represent metastatic colon cancers while *Apc*-only mutant organoids model benign tumors, these findings suggest that trogocytic activity is strongly associated with tumor progression and carcinogenesis.

Cancer stem cells (CSCs) represent a subpopulation of tumor cells endowed with self-renewal capacity and enhanced tumor-initiating and metastatic potential (Reya et al., 2001; Batlle and Clevers, 2017; Kim, 2025). Emerging evidence indicates that CSC frequency increases during tumor progression and metastasis across multiple cancer types, including colorectal cancer (O’Brien et al., 2007; Ricci-Vitiani et al., 2007). Colorectal CSCs have been implicated in therapy resistance, relapse, and dissemination to distant organs, and their dynamic plasticity is considered a key driver of metastatic progression (Batlle and Clevers, 2017). However, whether CSCs exhibit distinct trogocytic behavior compared with non-stem cancer cells (non-CSCs) has not been investigated.

Here, we directly compared trogocytic activity between CSCs and non-CSCs using Caco-2, HCT116 and HT-29-derived spheroids. We demonstrate that CSCs exhibit elevated trogocytosis compared with their non-stem counterparts. Moreover, we identify reduced expression of cholesterol 25-hydroxylase (CH25H) as a defining feature of CSCs. CH25H is a key enzyme involved in oxysterol metabolism and interferon-regulated cholesterol homeostasis (Blanc et al., 2013; Robertson and Ghazal, 2016; Lu et al., 2022). Our data show that decreased CH25H expression is associated with increased membrane fluidity, providing a mechanistic link between plasma membrane biophysics and trogocytic activity. Finally, integration with human colorectal cancer single-cell RNA sequencing data supports the clinical relevance of this regulatory axis. These findings reveal that a CH25H-low, membrane-fluidity-high phenotype defines a trogocytosis-active state in colon cancer stem cells.

## Methods

### Cell culture

Human colorectal cancer cell lines (Caco-2, HCT116 and HT-29) were maintained in complete DMEM (CM-DMEM) consisting of Dulbecco’s Modified Eagle Medium (DMEM) supplemented with 10% fetal bovine serum (FBS) and 1% penicillin-streptomycin. Cells were cultured in 100-mm culture dishes at 37 °C in a humidified incubator with 5% CO_2_. Culture medium was replaced every 2 days. During medium exchange, cells were gently washed with phosphate-buffered saline (PBS) to remove residual serum and cellular debris before fresh medium was added. Caco-2, HCT116 and HT-29 cells were routinely passaged every 3–4 days when they reached approximately 80%–90% confluence using trypsin-EDTA. For spheroid experiments, cells were expanded under adherent conditions and used when cell viability exceeded 98%. Jurkat T cells were maintained in suspension culture in RPMI-1640 medium supplemented with 10% FBS and 1% penicillin-streptomycin at 37 °C in a humidified incubator with 5% CO_2_. Cells were maintained at logarithmic growth density by periodic dilution with fresh medium.

### Three-dimensional spheroid culture

Caco-2, HCT116 and HT-29 spheroids were generated using two Matrigel-based three-dimensional (3D) culture formats depending on the experimental objective: a Matrigel bed method for functional analysis of trogocytosis and a Matrigel dome method for enrichment of CSCs populations (Supplementary Figure 1A) (Clevers, 2016; Samy et al., 2019). Adherent Caco-2, HCT116 and HT-29 cells were dissociated into single cells using trypsin-EDTA and resuspended in spheroid culture medium. Cell suspensions with viability greater than 98% were used for subsequent experiments. The spheroid medium consisted of a 1:1 mixture of Advanced DMEM/F-12 and CM-DMEM containing low glucose and 5% fetal bovine serum (FBS), supplemented with B-27 (1%) and penicillin-streptomycin (1%) (Supplementary Figure 1B). Matrigel (Corning® Matrigel® Growth Factor Reduced Basement Membrane Matrix, phenol red-free, LDEV-free, Corning Cat. No. 356231) was mixed with spheroid medium at a 1:1 ratio and dispensed into round-bottom 96-well plates (50 μL per well). After polymerization at 37 °C for 30 min, cells were seeded onto the Matrigel layer at a density of 2 × 10^3^ cells per well in 200 μL spheroid medium. This format was used for experiments designed to evaluate trogocytosis, as it allowed uniform spheroid formation and efficient recovery of cells for flow cytometry analysis. For the Matrigel dome method, cells were mixed with Matrigel at a 1:1 ratio and plated as dome structures at a density of 3 × 10^3^ cells per dome. After gelation at 37 °C for 30 min, spheroid medium was gently added to cover the domes. This culture condition was used to promote stable 3D growth and to increase the proportion of CSC populations. Cultures were maintained at 37 °C in an incubator with 5% CO_2_ and the media was replaced every 2 days. Under these conditions, cells progressively aggregated and formed compact spheroid structures, which were used for downstream analyses at the indicated time points.

### DiD labeling of Jurkat T cells

Jurkat T cells were labeled with DiD at a ratio of 5 μL per 1 × 10^6^ cells. The cell suspension was incubated in a 37 °C water bath for 15 min. After labeling, cells were washed with PBS and resuspended in either CM-DMEM or spheroid culture medium depending on the subsequent co-culture condition.

### Co-culture conditions

DiD-labeled Jurkat T cells were added at a 5:1 ratio (Parental or spheroid cells:Jurkat T cells). Prior to co-culture, the existing medium in each well was carefully removed to minimize dilution. Jurkat T cells prepared at the calculated ratio were then added directly to the wells, and co-culture was maintained for 24 h at 37 °C in an incubator with 5% CO_2_. For signaling analysis experiments, Jurkat T cells were added without DiD labeling to avoid potential interference from membrane dyes during downstream staining and fluorescence detection. In these experiments, Caco-2 spheroids and Jurkat T cells were co-cultured at a 1:1 ratio for 24 h prior to flow cytometric analysis.

### Transwell co-culture assay

To evaluate contact-dependent trogocytosis, transwell co-culture experiments were performed using Transwell permeable supports (0.4 μm pore size, Corning, Cat. No. 3412). Caco-2 spheroids were generated using a Matrigel bed method adapted for the transwell system. Briefly, Matrigel was mixed with spheroid culture medium at a 1:1 ratio and dispensed into each well (1.1 mL per well), followed by polymerization at 37 °C for 30 min. Caco-2 cells were then seeded onto the Matrigel layer at a density of 5 × 10^4^ cells per well and cultured for 6 days to allow spheroid formation.

DiD-labeled Jurkat T cells were prepared as described above and seeded into the upper transwell insert at a 5:1 ratio relative to Caco-2 spheroid cells. Co-culture was maintained for 24 h at 37 °C in a humidified incubator with 5% CO_2_. This configuration physically separated Jurkat T cells from spheroids while allowing exchange of soluble factors. Cells were subsequently harvested and processed for flow cytometric analysis as described above.

### Flow cytometry analysis

Cells were harvested after co-culture for flow cytometric analysis. Jurkat T cells in suspension were first collected into 15 mL conical tubes. For spheroid cultures, Matrigel (bed or dome) matrices were mechanically disrupted using culture medium followed by PBS washing to recover all cells. Cell pellets were obtained by centrifugation and dissociated with TrypLE for 10 min at 37 °C, with vortexing every 5 min. Cells were washed with PBS and resuspended for staining. For surface staining, cells were incubated for 30 min at 4 °C in the dark with the following antibodies: CD44 monoclonal antibody (IM7), eFluor™ 450 (eBioscience) at 1:80 dilution and CD133 (Prominin-1) monoclonal antibody (13A4), PE (eBioscience) at 1:20 dilution. In experiments requiring spectral compatibility with membrane probes, CD133 (Prominin-1) monoclonal antibody (TMP4), APC (eBioscience) was used instead of PE. For intracellular detection of LGR5, surface staining was performed first, followed by fixation and permeabilization using the True-Nuclear™ Transcription Factor Buffer Set (BioLegend, Cat. No. 424401). Cells were fixed in 1× fixation buffer for 20 min at room temperature, washed twice with PBS, and incubated in 1× perm buffer containing Fc block for 5 min at room temperature. Cells were then stained with anti-LGR5 mouse monoclonal antibody (OTIA2), Daylight 488 conjugate at 1:200 dilution for 20 min at 4 °C in the dark, washed, and resuspended in PBS for acquisition. For detection of phosphorylated STAT1 (Tyr701), cells were stained with rabbit monoclonal anti-phospho-STAT1 antibody (clone 1F23, Sigma-Aldrich, Cat. No. ZRB1520) at 1:200 dilution, followed by Alexa Fluor 647-conjugated goat anti-rabbit IgG (H + L) secondary antibody (Thermo Fisher Scientific, Cat. No. A21244) at a final dilution of 1:2000. To assess trogocytosis, Jurkat T cells were labeled with the lipophilic dye DiD. DiD was added at 5 µL per 1 × 10^6^ cells, diluted in PBS, and incubated for 15 min at 37 °C. Labeled cells were washed with PBS and used for co-culture experiments. Membrane lipid order was assessed using the polarity-sensitive probe di-4-ANEPPDHQ (Invitrogen, Thermo Fisher Scientific, Cat. No. D36802). Cells were stained with 0.25 μM di-4-ANEPPDHQ for 7 min at room temperature in the dark and immediately analyzed by flow cytometry. Fluorescence was collected in the B585 channel (high-order membrane state) and the B610 channel (low-order membrane state). All samples were analyzed on a flow cytometer after singlet gating, and marker-positive populations and dye transfer were quantified using FlowJo software.

### Membrane fluidity measurement by di-4-ANEPPDHQ

Membrane lipid order was evaluated using the polarity-sensitive fluorescent probe di-4-ANEPPDHQ (Invitrogen, Cat. No. D36802) (Jin et al., 2006; Waddington et al., 2019). Cells were harvested from 2D cultures or spheroids and washed once with PBS. The cell pellets were resuspended in PBS containing 0.25 μM di-4-ANEPPDHQ and incubated for 7 min at room temperature in the dark. After staining, cells were immediately analyzed by flow cytometry without fixation. The dye exhibits a spectral shift depending on membrane lipid packing, allowing discrimination between ordered and disordered membrane states (Jin et al., 2006). Fluorescence signals were collected using the B585 channel (high-order/ordered membrane) and the B610 channel (low-order/disordered membrane). Membrane lipid order was quantified by calculating the generalized polarization (GP) value for each population as previously described (Jin et al., 2006). GP was calculated from the geometric mean fluorescence intensity of the two channels using the following formula:

$$GP=\frac{I_{B585}-I_{B610}}{I_{B585}+I_{B610}}$$

where $I_{B585}$ and $I_{B610}$ represent the geometric mean fluorescence intensity in the B585 (ordered) and B610 (disordered) channels, respectively. GP values range from −1 to +1, with higher GP values indicating more ordered (less fluid) membranes. For CSC-specific analysis, cells were co-stained with anti-CD133 antibody (Prominin-1) Monoclonal Antibody (TMP4, APC, eBioscience) prior to di-4-ANEPPDHQ acquisition, and GP values were calculated within gated populations.

### RNA isolation and quantitative PCR

Total RNA was isolated from spheroids using the miRNeasy Mini Kit (QIAGEN, Cat. No. 217004) according to the manufacturer’s instructions. Cells were lysed in QIAzol Lysis Reagent (QIAGEN) and phase separation was performed using chloroform, followed by column-based RNA purification. RNA concentration and purity were measured using a DS-11 FX microvolume spectrophotometer (DeNovix Inc.). For complementary DNA (cDNA) synthesis, 1 μg of total RNA was reverse-transcribed using AccuPower® CycleScript RT PreMix (dT20) (Bioneer) according to the manufacturer’s protocol. Quantitative real-time PCR (qPCR) was performed using Dyne qPCR 2× PreMIX (SYBR Green with low ROX) (DyneBio) on a CFX Opus 96 Real-Time PCR System (Bio-Rad). Gene expression levels were analyzed using gene-specific primers for target genes, and *HPRT* was used as housekeeping control. Relative gene expression levels were calculated using the comparative Ct method as previously described (Taylor et al., 2019). Briefly, the threshold cycle difference between the target gene and *HPRT* was determined as:

$$\Delta Ct=Ct_{target}-Ct_{HPRT}$$

Relative expression levels were then calculated as:

$$\text{Relative }expression=2^{-\Delta Ct}$$

and presented as expression normalized to *HPRT*.

Quantitative PCR reactions were performed in four technical replicates for each target gene. Data are presented as mean ± standard deviation (SD). Statistical analyses were performed using GraphPad Prism software (version X; GraphPad Software). Differences between CSCs and non-CSCs were evaluated using a two-tailed unpaired Student’s t-test, and a p-value <0.05 was considered statistically significant.

### Single-cell RNA-sequencing data analysis

Publicly available single-cell RNA-sequencing data were obtained from the Genome Sequence Archive (GSA) under accession code HRA007643 and analyzed in this study. Filtered gene-barcode matrices from primary human colorectal cancer samples were imported into R and analyzed using Seurat (version 5.2.1) (Hao et al., 2024). Expression matrices were read using Read10X and converted into Seurat objects with Create Seurat Object, requiring at least three cells per gene and 200 features per cell. Individual samples were merged into a unified object for downstream analysis. Quality control filtering was performed based on the number of detected genes and mitochondrial transcript percentage. Cells with mitochondrial RNA content exceeding a sample-specific threshold (median +3 × MAD, capped at 30%) were removed. Cells were further filtered to retain those with 300–8,000 detected genes, and potential multiplets were excluded using sample-specific cutoffs for total UMI counts. Data were normalized using log-normalization, and the top 2,000 highly variable genes were identified using the “vst” method. During data scaling, mitochondrial transcript percentage was regressed out. Dimensionality reduction was performed using principal component analysis (PCA), and the first 12 principal components were used for neighbor graph construction, clustering, and visualization using Uniform Manifold Approximation and Projection (UMAP) (McInnes et al., 2018). Cell type annotation was performed based on canonical marker genes representing epithelial/tumor, immune, stromal, and endothelial lineages. Tumor epithelial cells were subsequently subset for further analysis. Developmental potential within the tumor epithelial population was estimated using CytoTRACE2 (version 1.1.0) applied to raw count matrices (Kang et al., 2025). The subset was reprocessed including normalization, variable feature detection, scaling, PCA, clustering, and UMAP visualization. To correct for inter-sample batch effects, data integration was performed using the Harmony R package with sample identity as the integration variable (version 1.2.3) (Korsunsky et al., 2019). Harmony-corrected embeddings were used for neighbor detection, clustering, and visualization. Stem-like tumor cells were inferred based on CytoTRACE2 scores and stemness marker expression. Cells were stratified into CH25H-high and CH25H-low groups based on the median CH25H expression level within each analyzed subset. Module scores were calculated using Seurat’s Add Module Score function for gene programs associated with stemness, WNT signaling, STAT1/interferon signaling, and lipid synthesis. Statistical significance for comparisons of gene expression and module scores between groups was assessed using the two-sided Wilcoxon rank-sum test.

## Results

### 3D spheroid culture transiently enriches a cancer stem cell population in Caco-2 cells

**FIGURE 1:**
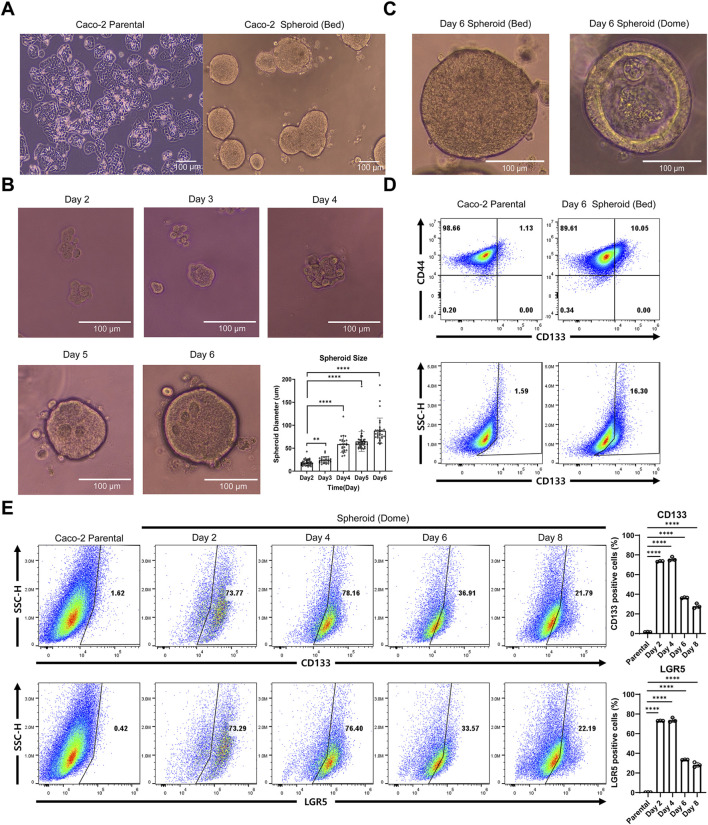
Increased cancer stem cell population in Caco-2 spheroids compared with 2D culture. **(A)** Representative bright-field images of Caco-2 cells cultured under conventional 2D conditions and 3D spheroid culture using the Matrigel bed format. Images show parental Caco-2 monolayer cells and day-6 spheroids formed under 3D culture conditions. Scale bars, 100 μm. **(B)** Time-course images show progressive spheroid formation following single-cell dissociation during 3D culture (day 2–6). Quantification of spheroid diameter over time shows progressive spheroid enlargement. Statistical significance was determined using mixed effects analysis followed by Dunnett’s multiple comparisons test. **(C)** Representative bright-field images comparing spheroids generated using Matrigel bed and dome culture formats. **(D)** Flow cytometry analysis of CD44 and CD133 expression in bed-derived spheroids compared with parental cells shows increased CD44^+^CD133+ CSC population under spheroid culture conditions. **(E)** Time-dependent flow cytometry analysis of the CSC marker CD133 and LGR5 during dome spheroid culture. Statistical significance was determined using one-way ANOVA followed by Dunnett’s multiple comparisons test. Data are presented as mean ± SD from independent experiments. Spheroid diameter analysis **(B)** was performed by measuring 24–40 individual spheroids per time point, and flow cytometry analyses **(D,E)** were performed from three independent experiments (n = 3). *p < 0.05, **p < 0.01, ***p < 0.001, ****p < 0.0001.

Previous studies have demonstrated that 3D spheroid culture enriches CSC populations relative to conventional 2D culture (Weeber et al., 2015; Clevers, 2016). To establish an experimental system for comparing trogocytosis between CSCs and non-CSCs, we first generated Caco-2 spheroids under optimized 3D culture conditions (Supplementary Figures 1A,B). In contrast to 2D-cultured Caco-2 cells, dissociated cells cultured in 3D readily aggregated to form compact multicellular spheroids (Figure 1A). Time-course imaging revealed progressive spheroid formation accompanied by a significant increase in spheroid diameter (Figure 1B). By day 5, spheroids displayed a well-organized architecture characterized by a distinct outer epithelial layer and detached dying cells in the interior. Notably, this structural organization was reproducibly observed under both Matrigel bed and dome culture conditions (Figure 1C).

We then examined whether spheroid culture enriches CSC populations. Flow cytometry analysis using established colorectal CSC markers including CD44 and CD133, revealed that 3D-cultured Caco-2 spheroids exhibited an approximately 10-fold enrichment of the CSC population at day 6 compared with 2D-cultured parental Caco-2 cells (Figure 1D). To further characterize the temporal dynamics of stem-like cells during spheroid formation, we monitored the expression of CD133 and LGR5 over time. Given that CD44 expression was uniformly positive across all tumor cells under our culture conditions, LGR5 was employed as an alternative CSC marker to better distinguish stem-like subpopulations. During the early phase of spheroid formation, both markers were progressively upregulated with approximately 70%–75% of cells expressing each marker at day 2–4. These were followed by a gradual decline as the spheroids matured through days 6–8 (Figure 1E). This transient enrichment pattern is consistent with our previous observations in primary murine colon cancer organoids harboring *Apc*, *Kras* and *Tp53* mutations, in which LGR5 expression increased shortly following replating of dissociated cells and declined at later stages (Shin et al., 2021a). Collectively, these results demonstrate that 3D spheroid culture transiently enriches a CSC population in Caco-2 cells. Based on this temporal expression pattern, day 4–6 spheroids, in which CSC and non-CSC populations coexist, were selected for subsequent experiments.

### Cancer stem cells in Caco-2 spheroids exhibit enhanced trogocytosis

**FIGURE 2:**
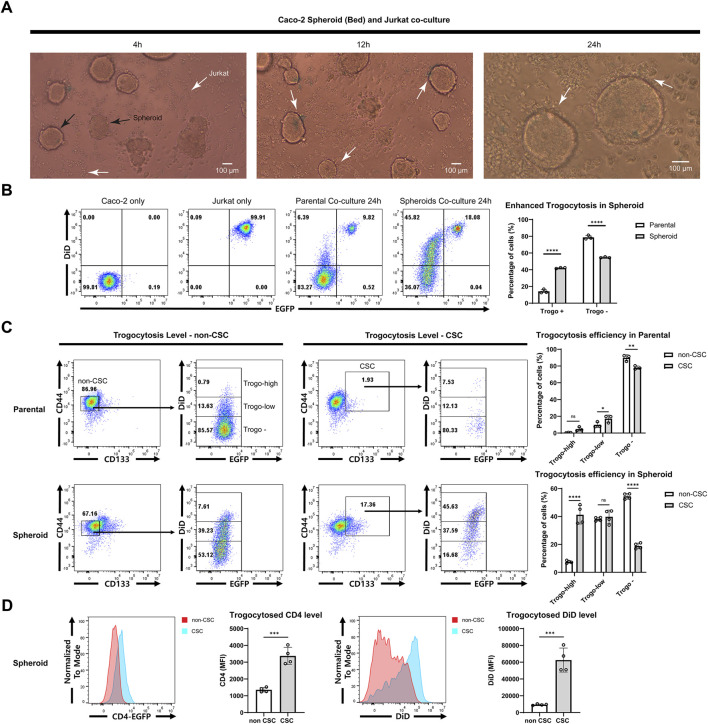
Enhanced trogocytosis of colon cancer stem cells in spheroids. **(A)** Representative bright-field images of Caco-2 spheroids co-cultured with Jurkat T cells for 4, 12, and 24 h. Jurkat T cells progressively accumulated around spheroids over time (white arrows, Jurkat T cells; black arrows, spheroids). Scale bars, 100 μm. **(B)** Flow cytometry analysis of membrane transfer after 24 h co-culture. DiD-labeled, CD4-EGFP-expressing Jurkat T cells were co-cultured with 2D-cultured parental Caco-2 cells or 3D spheroid-derived Caco-2 cells. Control samples including Caco-2 only cells and DiD-labeled CD4-EGFP-expressing Jurkat T cells were used to define background fluorescence and gating. Representative plots and quantification of trogocytosis-positive (Trogo+) and trogocytosis-negative (Trogo−) populations are shown. Statistical significance was determined using unpaired two-tailed Student’s *t*-tests. **(C)** Trogocytosis analysis in non-CSC and CSC populations from parental cells and spheroids. Cells were first gated as non-CSC or CSC based on CD44 and CD133 expression, and trogocytosis levels were then further classified according to transferred CD4-EGFP and DiD signals as Trogo-high, Trogo-low, and Trogo− populations. Representative plots and quantification are shown. Statistical significance was determined using two-way ANOVA followed by Sidak’s multiple comparisons test. **(D)** Histogram overlays and quantification of mean fluorescence intensity (MFI) of trogocytosed CD4-EGFP and DiD in non-CSC and CSC populations from spheroids. Statistical significance was determined using unpaired two-tailed Student’s *t*-tests. Data are presented as mean ± SD from independent experiments. **(B)** was analyzed from three independent experiments (n = 3), **(C)** from parental (n = 3) and spheroid (n = 4) samples, and **(D)** from four independent experiments (n = 4). *p < 0.05, **p < 0.01, ***p < 0.001, ****p < 0.0001; ns, not significant.

To quantify trogocytosis-mediated membrane transfer, we established a Jurkat T cell clone stably expressing a CD4-EGFP fusion protein (Supplementary Figure 2A). Jurkat T cells were additionally labeled with the membrane dye DiD to test overall membrane acquisition. These labeled Jurkat T cells were co-cultured with either parental Caco-2 monolayers or 3D spheroids, and trogocytosis was assessed by flow cytometry as described previously. Bright-field imaging showed time-dependent accumulation of Jurkat T cells around spheroids from 4 to 24 h, with extensive attachment to spheroid surfaces at later time points consistent with active cell-to-cell interactions (Figure 2A). Flow cytometry analysis after 24 h of co-culture revealed markedly greater trogocytosis in spheroids compared with 2D-cultured parental Caco-2 cells (Figure 2B). Approximately 55% of spheroid-derived Caco-2 cells were trogocytosis-positive (CD4-EGFP + DiD+), compared with approximately 10% of parental cells. To confirm that trogocytosis requires direct cell-to-cell interactions, we performed co-culture experiments using a transwell system. Under these conditions, trogocytosis in 3D spheroids was abolished, indicating that membrane transfer occurs through direct cell-to-cell contact (Supplementary Figure 2B). Together, these findings demonstrate that 3D spheroid culture markedly enhances contact-dependent trogocytosis.

We next asked whether this increase was preferentially associated with cancer stem cells (CSCs). The enrichment of CSCs in spheroids relative to parental cultures was preserved during co-culture (Supplementary Figure 2C). After 24 h co-culture, CSCs and non-CSCs were identified based on CD44 and CD133 expression. Trogocytosis levels of CSCs and non-CSCs were classified into Trogo-high, Trogo-low and Trogo-negative populations based on the acquired CD4-EGFP and DiD signals from Jurkat T cells (Figure 2C). In 2D parental cultures, CSCs showed only modestly higher trogocytosis than non-CSCs. In spheroids, this difference became more pronounced. Spheroid-derived CSCs were predominantly in the Trogo-high population, whereas non-CSCs were mostly in the Trogo-low or Trogo-negative populations. Consistent with this observation, CSCs contained higher levels of trogocytosed CD4-EGFP and DiD than non-CSCs (Figure 2D). Enhanced trogocytic activity of CSCs is further confirmed using HCT116 and HT-29-derived spheorids (Supplementary Figures 3A,B). Collectively, these results show that 3D spheroid culture not only increases overall trogocytosis but also preferentially enriches a trogocytosis-active CSC subpopulation, suggesting that enhanced membrane acquisition is a functional feature of CSCs. TRANSWELL.

### CSCs in colorectal cancer spheroids exhibit increased membrane fluidity

**FIGURE 3:**
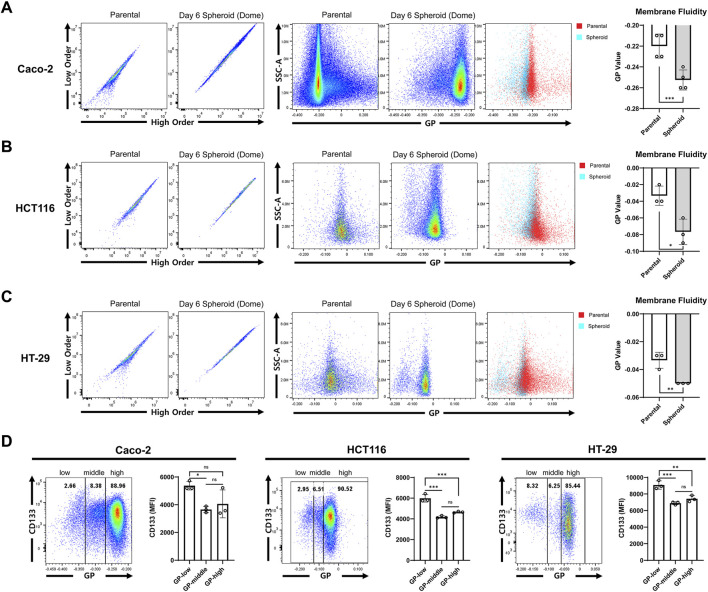
Membrane fluidity is increased in CD133+ stem cells within colon cancer spheroids. **(A–C)** Membrane lipid order was analyzed by flow cytometry using the polarity-sensitive probe di-4-ANEPPDHQ in 2D-cultured parental cells and day 6 Matrigel dome-cultured spheroids derived from Caco-2 **(A)** HCT116 **(B)** and HT-29 **(C)** cells. Representative high-order and low-order fluorescence plots are shown. Membrane lipid order was quantified using generalized polarization (GP) values. Statistical significance was determined using an unpaired two-tailed Student’s t-test. **(D)** Spheroid-derived cells from Caco-2, HCT116 and HT-29 cultures were stratified into GP-low, GP-middle and GP-high populations, and CD133 levels were analyzed in each population. Statistical significance was tested using one-way ANOVA followed by Tukey’s multiple-comparisons test. Results are representative of at least three independent experiments. *p < 0.05, **p < 0.01, ***p < 0.001; ns, not significant; MFI, mean fluorescence intensity.

We next asked whether membrane biophysical properties distinguish CSCs from non-CSCs. Because membrane lipid organization influences membrane deformation and intercellular membrane exchange, we assessed membrane order using the polarity-sensitive probe di-4-ANEPPDHQ (Simons and Ikonen, 1997; Janes et al., 2000; Jin et al., 2006; Lingwood and Simons, 2010; Waddington et al., 2019). Under the optimized staining condition (0.25 μM), membrane order was compared between 2D cultured parental Caco-2 cells and 3D spheroids (Supplementary Figures 4A,B). Spheroid-derived cells showed a clear shift toward the low-order fluorescence state relative to parental cells (Figure 3A), indicating the emergence of a distinct low-order membrane population forming a separate fluorescence band above the main population. Consistent with this observation, generalized polarization (GP) analysis revealed a significant decrease in GP values in spheroids, indicating increased membrane fluidity under the 3D culture condition (Figure 3A). Similarly, HCT116-derived and HT-29-derived spheroids showed increased membrane fluidity compared to their 2D-cultured parental cell lines (Figures 3B,C). We next examined whether this altered membrane state was associated with CSCs. To address this, spheroid cells were classified according to GP-defined membrane states (GP-low, GP-middle, and GP-high) and CD133 expression was analyzed within each population. In all three spheroid models, CD133 expression was significantly higher in GP-low cells, defined by reduced membrane order, than in the GP-middle or GP-high cells (Figure 3D). These results demonstrate that 3D culture increases membrane fluidity and CSCs preferentially occupy a high-fluidity membrane state.

### Reduced CH25H expression and attenuated STAT1 signaling characterize a trogocytosis-active CSC phenotype

**FIGURE 4:**
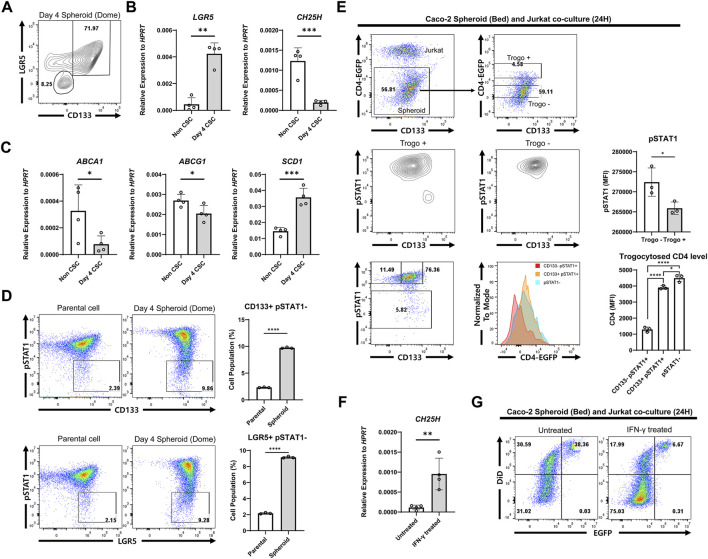
Reduced CH25H expression and attenuated STAT1 signaling are associated with lipid metabolic remodeling in spheroid-derived CSCs. **(A)** Representative flow cytometry plots showing the sorting strategy used to isolate CSC and non-CSC populations from day-4 Caco-2 spheroids based on CD133 and LGR5 expression. **(B)** Quantitative RT-PCR analysis of *LGR5* and *CH25H* expression in sorted CSC and non-CSC populations. **(C)** Quantitative RT-PCR analysis of lipid metabolism-related genes in sorted populations. Expression levels of *ABCA1*, *ABCG1* and *SCD1*. All gene expression levels were normalized to *HPRT*. **(D)** Flow cytometry analysis of STAT1 signaling. Representative plots show pSTAT1 staining in CD133+ or LGR5+ populations from 2D parental cultures and spheroid cultures. Statistical significance was determined using one-way ANOVA followed by Tukey’s multiple comparisons test. **(E)** Trogocytosis analysis in cells stratified by STAT1 phosphorylation level. Flow cytometry plots and quantification of trogocytosed CD4 signal in pSTAT1-low and pSTAT1-high populations are shown. Statistical significance was determined using unpaired two-tailed Student’s t-test. **(F)** Quantitative RT-PCR analysis of *CH25H* expression in Caco-2 spheroids treated with recombinant human IFN-γ (1,000 U/mL, 24 h). Gene expression levels were normalized to *HPRT*. Statistical significance in **(B,C,F)** was determined using unpaired two-tailed Student’s t-test. **(G)** Trogocytosis analysis following IFN-γ treatment. Representative flow cytometry plots (DiD vs. EGFP) showing membrane transfer and quantification of Trogo+ and Trogo− populations in untreated and IFN-γ treated spheroids are shown. Data are presented as mean ± SD from independent experiments. **(B,C,F)** were analyzed from four independent experiments (n = 4), **(D,E)** from three independent experiments (n = 3). *p < 0.05, **p < 0.01, ***p < 0.001, ****p < 0.0001; ns, not significant.

To investigate the molecular mechanisms underlying the membrane phenotype of CSCs, we isolated CSCs and non-CSCs from day 4 or day 10 Caco-2 spheroids based on CD133 and LGR5 expression (Figure 4A; Supplementary Figure 5A). Post-sorting flow cytometry confirmed efficient enrichment of the LGR5+CD133+ CSC population in spheroid cultures. Quantitative RT-PCR analysis further validated enrichment of *LGR5* and revealed significantly reduced *CH25H* expression in CSCs compared with non-CSCs (Figure 4B). Given that CH25H catalyzes cholesterol hydroxylation, its downregulation is expected to limit cholesterol turnover, contributing to the retention of cholesterol within cellular membranes (Robertson and Ghazal, 2016). Consistent with this, expression of the cholesterol efflux transporter *ABCA1* and *ABCG1* was reduced in CSCs, suggesting decreased cholesterol export (Figure 4C; Supplementary Figure 5B). In parallel, the lipogenic enzyme *SCD1* was significantly upregulated. *SCD1* catalyzes the conversion of saturated fatty acids into monounsaturated fatty acids, thereby modulating membrane lipid composition and biophysical properties. Previous studies have shown that *SCD1* is preferentially upregulated in colon and other cancer stem-like cells and is required for CSC maintenance, in part through regulation of Wnt/NOTCH-associated programs (Noto et al., 2013; Yu et al., 2021). Similar lipid metabolism-related gene expression patterns were observed in HCT116-dervied spheroids, further supporting this CSC-associated lipid metabolic signature (Supplementary Figure 5C). These results suggest that CSCs exhibit reduced cholesterol hydroxylation and efflux together with increased lipid desaturation, which may influence membrane cholesterol levels and associated membrane properties.

Because CH25H is a well-established interferon-responsive gene downstream of STAT1 signaling, we next assessed STAT1 activation by measuring STAT1 phosphorylation (pSTAT1) in spheroid cultures (Blanc et al., 2013; Magoro et al., 2019). Flow cytometry analysis revealed a marked increase in CSC marker-positive/pSTAT1-negative populations in day 4 spheroids compared with parental cells including both CD133+pSTAT1-and LGR5+pSTAT1-cells (Figure 4D). These results indicate reduced STAT1 signaling in CSC-enriched spheroids. To determine the relationship between STAT1 signaling and membrane acquisition, we analyzed trogocytosis in cells stratified by membrane uptake following co-culture with Jurkat T cells. Cells within the Trogo-high population exhibited significantly lower pSTAT1 levels than cells in the Trogo-negative population (Figure 4E). Consistently, CD133+pSTAT1-cells displayed higher levels of trogocytosed CD4-EGFP proteins compared to pSTAT1+ cells. Furthermore, IFN-γ treatment increased *CH25H* expression in spheroids while reducing the proportion of CD133+LGR5+ CSCs (Figure 4F; Supplementary Figure 5D). IFN-γ induces STAT1 phosphorylation in Caco-2 cells within 24 h (Magro et al., 2005). Consistent with this, IFN-γ treatment also reduced trogocytosis in Caco-2 spheroids in the 3D co-culture system (Figure 4G). These results support an inverse relationship between STAT1 signaling and trogocytic membrane acquisition. Collectively, spheroid-derived CSCs exhibit reduced CH25H expression and STAT1 signaling, accompanied by enhanced trogocytosis.

### Reduced STAT1 signaling and CH25H downregulation in human colorectal cancer patient-derived single cell RNA-sequencing data

**FIGURE 5:**
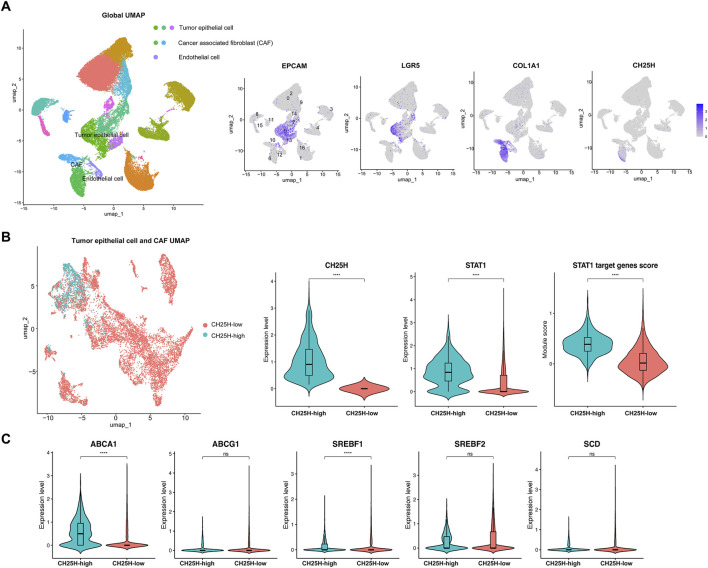
Reduced CH25H expression and STAT1 signaling in human colorectal cancer single-cell analysis **(A)** UMAP visualization of single-cell transcriptomes from primary human colorectal cancer (CRC) samples (GSA: HRA007643). Major cellular compartments were identified based on canonical markers including tumor epithelial cells (EPCAM, LGR5), cancer-associated fibroblasts (COL1A1), endothelial cells and immune cells. Feature plots show the expression of *EPCAM*, *LGR5*, *COL1A1* and *CH25H* across the global cell populations. **(B)** Tumor epithelial cells and CAFs were isolated together and clustered by UMAP. Cells were stratified into *CH25H-high* and *CH25H-low* populations. Violin plots show the expression of *CH25H*, *STAT1*, and STAT1 target gene module scores. **(C)** Violin plots showing the expression of selected lipid-related genes (*ABCA1*, *ABCG1*, *SREBF1*, *SREBF2*, and *SCD*) in *CH25H*-low versus CH25H-high cells within the tumor epithelial cell and CAF subset. Violin plots include boxplots, and statistical significance was determined using the Wilcoxon rank-sum test. *p < 0.05, **p < 0.01, ***p < 0.001, ****p < 0.0001; ns, not significant.

To test whether reduced CH25H expression is associated with decreased STAT1 signaling in patient-derived colorectal cancer (CRC) samples, we analyzed publicly available single-cell RNA-sequencing (scRNA-seq) data from primary CRC tumors (GSA: HRA007643). Uniform manifold approximation and projection (UMAP) analysis revealed clusters of tumor epithelial cells, cancer-associated fibroblasts (CAFs), endothelial cells, and immune cells based on canonical markers such as EPCAM, LGR5, and COL1A1 (Figure 5A; Supplementary Figures 6A,B). CytoTRACE2 analysis revealed heterogeneous cell states within tumor epithelial populations, with a subset of cells displaying high stemness scores and enriched expression of intestinal stem cell markers, including LGR5, PROM1, and CD44 (Supplementary Figures 6C,D). Consistent with our findings in Caco-2 spheroids, CSCs exhibited increased Wnt target gene expression and reduced STAT1 target gene scores, accompanied by a decreasing trend in CH25H expression (Supplementary Figures 7A,B). In CRC patient scRNA-seq data, CH25H expression was detected in both tumor epithelial and CAF populations (Figure 5A). These cell types were therefore selected for further analysis. CAF populations were stratified into CH25H-high and CH25H-low groups, and STAT1 expression as well as STAT1 target gene activity were compared between these groups. Consistent with the epithelial compartment, CH25H-low CAFs exhibited lower STAT1 expression and decreased STAT1 target gene module scores compared with CH25H-high CAFs (Supplementary Figures 7C,D). When tumor epithelial cells and CAFs were analyzed together, CH25H-low populations showed reduced STAT1 expression and STAT1 target gene scores (Figure 5B). In addition, reduced expression of ABCA1 and SREBF2 was observed in CH25H-low populations, consistent with findings in Caco-2 spheroids (Figures 4C, 5C). Collectively, these results support the presence of a conserved STAT1-CH25H signaling axis across tumor-associated cell populations in human colorectal cancer.

## Discussion

Trogocytosis has emerged as a key mechanism of intercellular communication in the tumor microenvironment that contributes to immune evasion (Joly and Hudrisier, 2003, Shin et al., 2021b). However, whether trogocytosis varies across distinct tumor cell states has remained unclear. In this study, we show that trogocytosis occurs preferentially in CSCs within Caco-2 spheroids. CSC-enriched spheroids exhibited greater membrane acquisition from Jurkat T cells via trogocytosis together with increased membrane fluidity, reduced CH25H expression, and coordinated changes in lipid metabolism-related genes. These findings identify a trogocytosis-acitve CSC phenotype characterized by low CH25H expression and increased membrane fluidity.

Our results extend previous observations linking tumor progression with increased trogocytosis. In our earlier work, genetically defined murine colon cancer organoids representing advanced disease showed higher trogocytic activity than less malignant counterparts. Here, we further show that this phenotype is not uniformly distributed across tumor cells, but is preferentially associated with the CSC state (Tape, 2024). Because CSCs are closely linked to tumor initiation, metastasis, therapy resistance and recurrence, the elevated trogocytosis may represent an additional functional feature of stem-like tumor cells (Cañellas-Socias et al., 2022; Ebrahimi et al., 2023; Tape, 2024). A major finding of this study is the association between CSC state and membrane biophysics. Spheroid cultures displayed lower membrane order than 2D parental cultures, consistent with increased membrane fluidity, and CSC-associated cells were enriched in the fluidity-high population. This suggests that the CSC membrane phenotype is better viewed as a biased biophysical state within a heterogeneous population rather than a fully discrete membrane-order class (Tape, 2024). A more fluid membrane would be expected to reduce the physical barrier to membrane deformation and fragment exchange, providing a plausible basis for enhanced trogocytosis in CSCs.

Our data identify CH25H as a potential regulator of membrane-associated features in CSCs. In sorted CSC populations, CH25H expression was reduced together with decreased expression of cholesterol efflux–related genes such as *ABCA1* suggesting alterations in lipid metabolic programs (Blanc et al., 2013; Robertson and Ghazal, 2016). Notably, this trend was broadly consistent with transcriptional patterns observed in independent colorectal cancer patient-derived single-cell RNA-sequencing data, where CH25H-low populations showed reduced expression of cholesterol efflux–associated genes such as *ABCA1* (Batlle and Clevers, 2017; Papadaki and Magklara, 2022). These changes may contribute to a more deformable membrane state (Simons and Ikonen, 1997; Lingwood and Simons, 2010). Importantly, increased membrane fluidity in CSCs is unlikely to be explained by changes in total cholesterol levels alone, but rather reflects coordinated remodeling of membrane lipid composition. Reduced CH25H expression would be expected to decrease 25-hydroxycholesterol (25HC) levels, thereby relieving oxysterol-mediated membrane stabilization. In parallel, increased SCD1 expression may promote lipid desaturation, leading to reduced lipid packing and increased membrane disorder. Together, these changes provide a plausible basis for the fluid membrane state observed in CSCs. Consistent with this, previous studies have shown that CH25H can modulate membrane dynamics and constrain trogocytosis in CAR-T cells (Blanc et al., 2013; Lu et al., 2022; Schoutrop et al., 2022). These findings suggest that reduced CH25H expression contributes to increased membrane fluidity, potentially through changes in oxysterol levels and lipid composition, thereby facilitating trogocytosis.

The signaling context of this phenotype further supports this interpretation. CSC-enriched spheroids contained more CSC marker-positive/pSTAT1-negative cells, and pSTAT1-low cells exhibited greater membrane transfer than pSTAT1-high cells. Because CH25H is a canonical interferon-stimulated gene downstream of STAT1 signaling, attenuation of interferon-STAT1 signaling may contribute to reduced CH25H expression in CSCs (Blanc et al., 2013; Schneider et al., 2014). Consistent with this possibility, interferon-gamma (IFN-γ) treatment in 3D culture showed elevation of *CH25H* expression in spheroids and their trogocytosis activity was reduced (Figures 4F,G). Moreover, analysis of human colon cancer single-cell RNA-seq datasets revealed a correlation between *CH25H* and *STAT1* expression, supporting the relevance of this signaling axis beyond the Caco-2 model (Aybey et al., 2025). These findings suggest that suppression of interferon-associated signaling and metabolic remodeling of lipid pathways may jointly contribute to the membrane phenotype observed in CSCs (Schneider et al., 2014; Padariya et al., 2021). CSCs are known to exhibit substantial metabolic plasticity, and enhanced lipid desaturation has been implicated in stemness maintenance and tumor progression (Papadaki and Magklara, 2022; Du and Qin, 2024; Lim, 2024). The increase in *SCD1* observed in this study is therefore consistent with the idea that stemness-associated lipid metabolism and reduced cholesterol-regulatory signaling together shape a membrane environment permissive for trogocytosis (Du and Qin, 2024).

Several limitations should be acknowledged. First, this study was designed to define a CSC-associated trogocytosis phenotype and identify candidate regulatory pathways rather than establish a complete causal hierarchy. Accordingly, although our data identify CH25H as a strong candidate regulator, direct gain- and loss-of-function studies are planned for future work to determine whether CH25H is necessary and/or sufficient to regulate membrane order and trogocytosis in tumor cells. Second, although our functional analyses were performed in three different cell line-derived spheroid models, validation in additional colorectal cancer systems including patient-derived organoids and primary tumor samples will be needed to assess the generalizability of our findings. Importantly, these limitations do not alter the central conclusion of the present study: a trogocytosis-active state is enriched within colon cancer CSCs and is associated with a distinct membrane and transcriptional phenotype.

In conclusion, this study identifies a previously unrecognized link between cancer stem cell state, membrane fluidity, and trogocytosis in colorectal cancer. CSC-enriched Caco-2 spheroids exhibited enhanced trogocytic membrane acquisition together with increased membrane fluidity, reduced CH25H expression, lipid metabolic remodeling, and diminished STAT1 signaling. These findings support a model in which membrane biophysical remodeling accompanies the stem-like tumor state and favors trogocytosis. More broadly, our results extend the study of trogocytosis beyond immune-centered frameworks and position membrane state as a mechanistically relevant feature of colon cancer stem cells.

## Data Availability

The original contributions presented in the study are included in the article/Supplementary Material, further inquiries can be directed to the corresponding author.
