# Supplementary material for: Reduced CH25H expression defines a high membrane fluidity, trogocytosis active state in colon cancer stem cells
Source: Front Cell Dev Biol. 2026 Jul 9;14:1834358. doi: 10.3389/fcell.2026.1834358 (PMC13391522; doi:10.3389/fcell.2026.1834358)
Supplement: Supplementary file 1 [file Supplementaryfile1.docx]

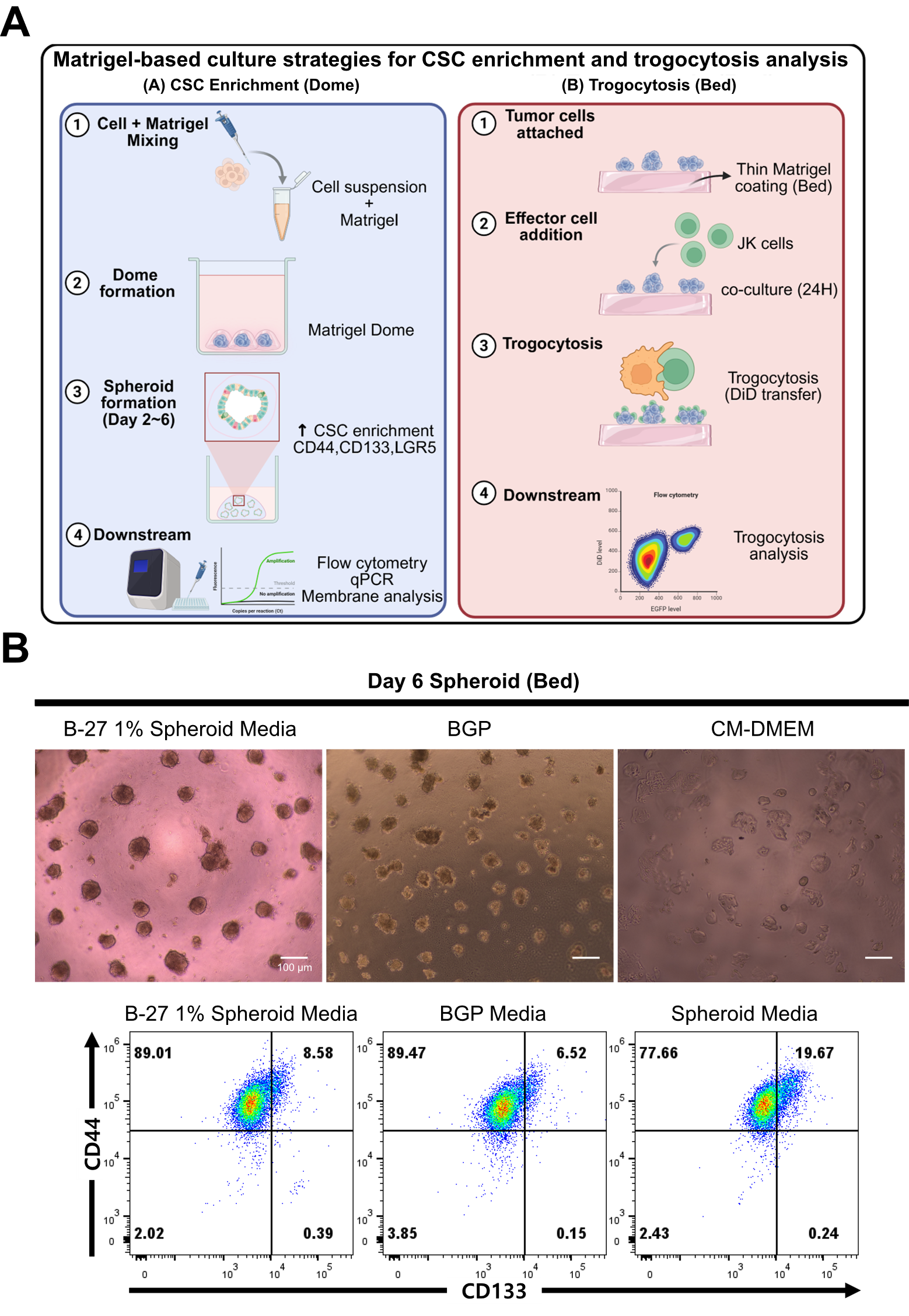


**Supplementary Figure 1. Matrigel-based spheroid culture strategy and medium conditions used for CSC enrichment.**

**(A)** Schematic illustration of the Matrigel-based culture strategies. Caco-2 cells were mixed with Matrigel and cultured in 3D dome for CSC enrichment. In order to co-culture tumor cells and T cells for trogocytosis analysis, Caco-2 cells were cultured on a thin Matrigel-coated plate (bed). Once spheroids formed, Jurkat T cells were added, then trogocytosis was analyzed by flow cytometry. **(B)** Evaluation of culture media conditions for spheroid formation. Representative images of spheroids cultured in B-27-supplemented spheroid medium and flow cytometry analysis of CSC markers (CD133 and CD44) in cells cultured under different media conditions.


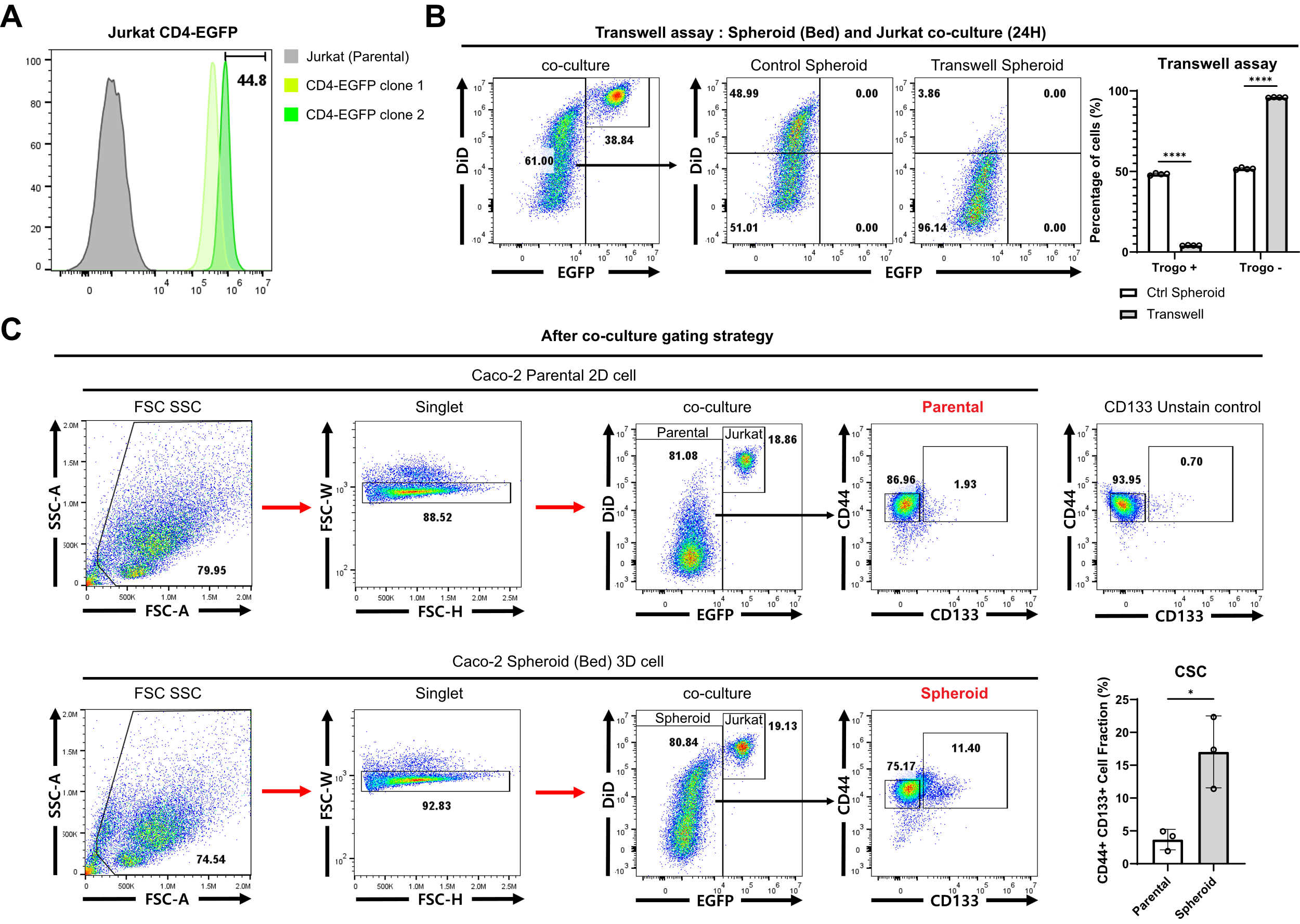


**Supplementary Figure 2. Generation of CD4-EGFP fusion protein expressing Jurkat clones for co-culture and co-culture gating strategy.**

**(A)** Flow cytometry showed CD4-EGFP expression in Jurkat T cells. Cloned Jurkat lines stably expressing CD4-EGFP were used for subsequent experiments. **(B)** Trogocytosis analysis in Caco-2 spheroids co-cultured with Jurkat T cells under direct contact (Ctrl spheroid) or transwell-separated conditions. Representative flow cytometry plots (DiD vs EGFP) and quantification of trogocytosis-positive (Trogo+) and trogocytosis-negative (Trogo−) populations are shown. **(C)** Representative gating strategy used for flow cytometric analysis after co-culture with Caco-2 2D parental cells or Caco-2 spheroids (bed culture). After FSC/SSC and singlet gating, Caco-2 and Jurkat populations were distinguished in the co-culture samples, and CSCs were identified as CD133+CD44+ cells. A CD133 unstained control was included to define the positive gate. Quantification of the CSC fraction is shown at right. Data are presented as mean ± SD. Trogocytosis comparisons between direct contact and transwell conditions **(B)** were analyzed using two-way ANOVA followed by Sidak’s multiple comparisons test (n = 4 independent experiments). CSC fraction comparisons **(C)** were analyzed using unpaired two-tailed Student’s *t*-test (n = 3 independent experiments). *p < 0.05, ****p < 0.0001.

**
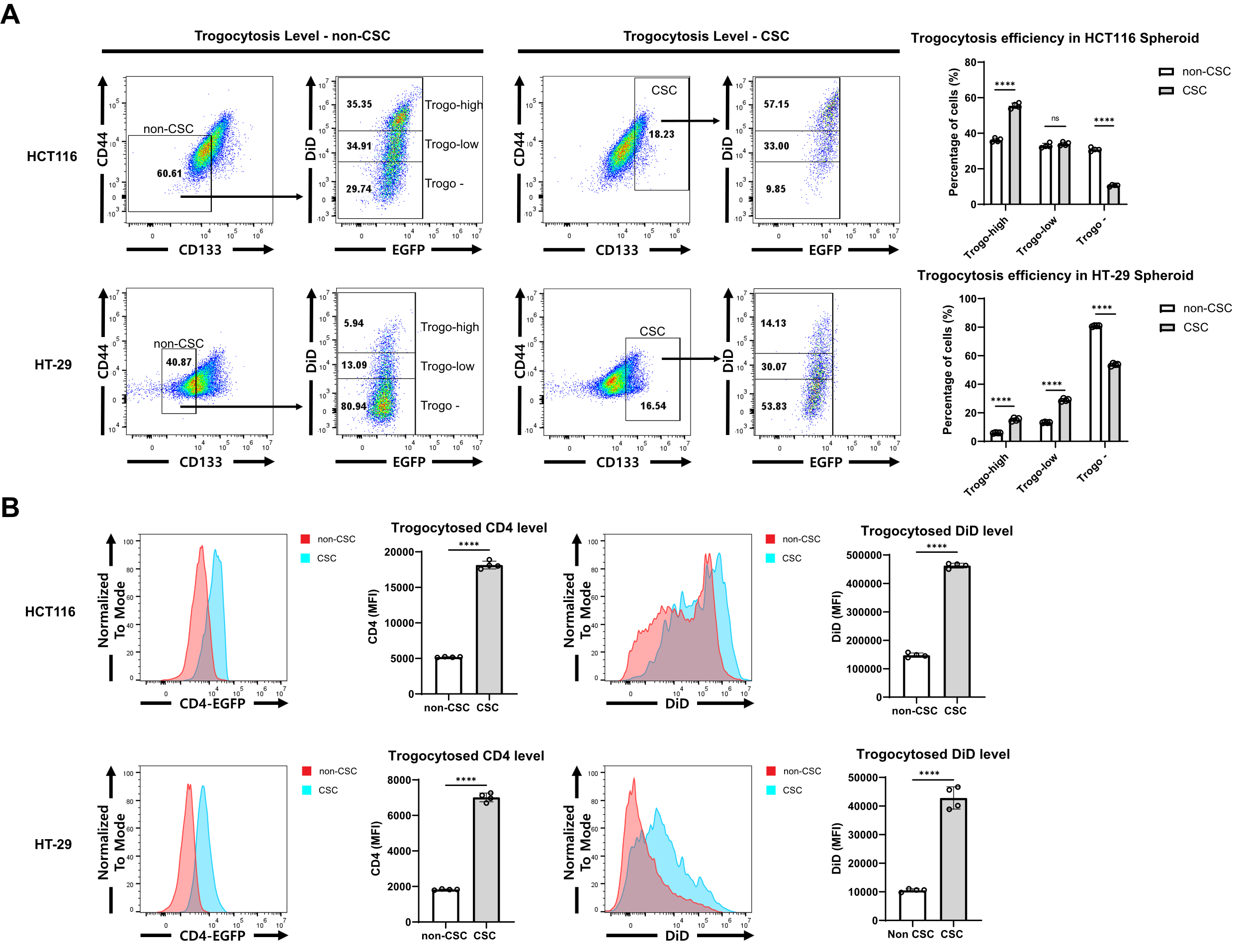
**

**Supplementary Figure 3. Enhanced trogocytosis of cancer stem cells in HCT116 and HT-29 spheroids.**

**(A)** Trogocytosis efficiency in non-CSC and CSC populations derived from HCT116 and HT-29 spheroids. Representative flow cytometry plots show the stratification into Trogo-high, Trogo-low, and Trogo negative (Trogo -) subsets. **(B)** Representative histograms and quantification of transferred CD4-EGFP and DiD signals in non-CSC versus CSC populations in HCT116 and HT-29 spheroids. Trogocytosis efficiency comparisons across subsets **(A)** were analyzed using two-way ANOVA followed by Sidak’s multiple comparisons test (n = 4 independent experiments). Mean fluorescence intensity (MFI) comparisons **(B)** were analyzed using unpaired two-tailed Student’s *t*-tests (n = 4 independent experiments). ***p < 0.001, ****p < 0.0001; ns, not significant.


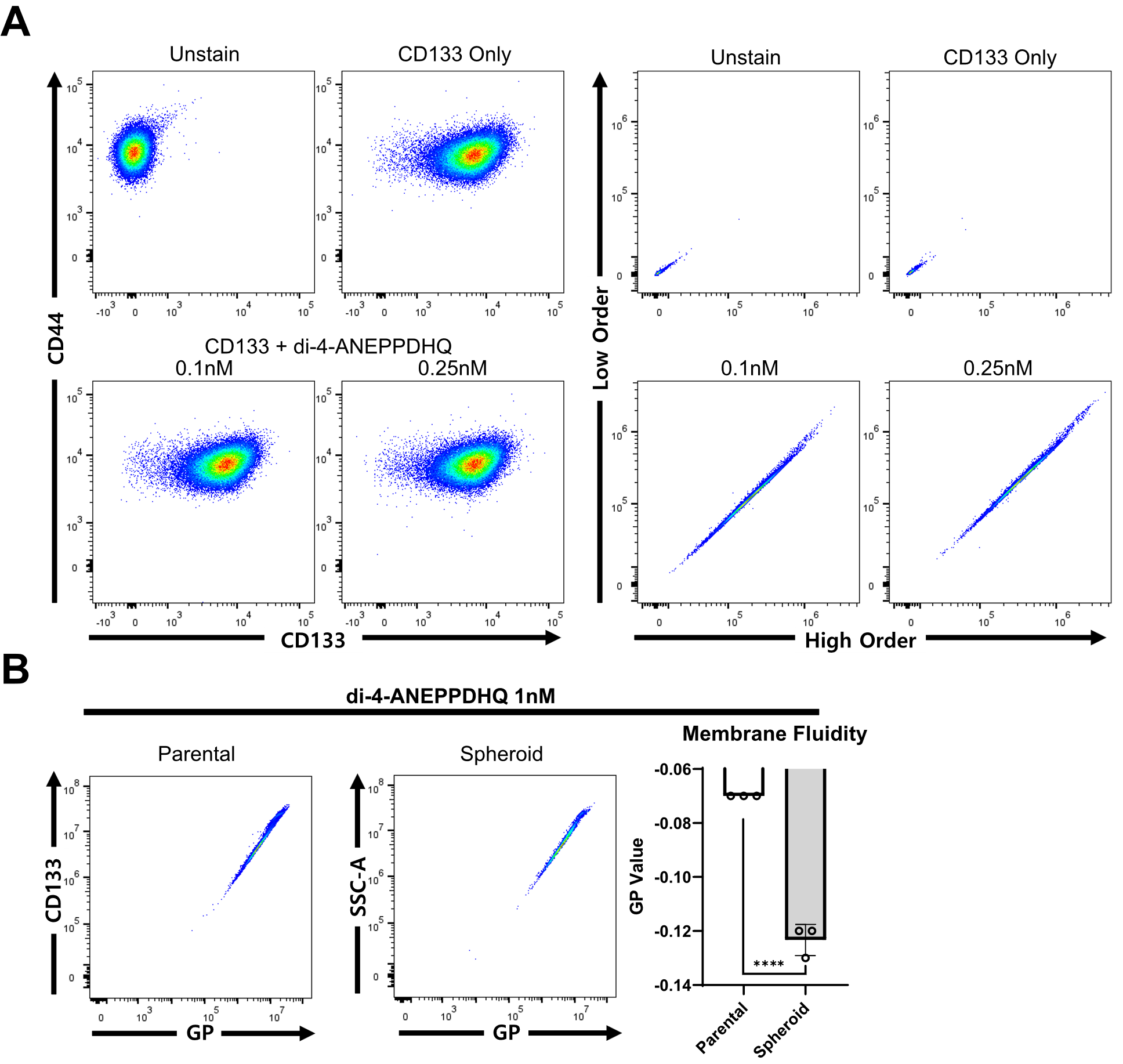


**Supplementary Figure 4. Optimization of di-4-ANEPPDHQ staining conditions for membrane order measurement.**

**(A)** Flow cytometry analysis performed to evaluate staining conditions for simultaneous detection of CD133 and membrane order using di-4-ANEPPDHQ. **(B)** Representative GP plots and quantification of membrane order in parental Caco-2 cells and spheroid cultures stained with di-4-ANEPPDHQ. GP values were calculated from the high-order and low-order emission channels. Data are presented as mean ± SD (n = 3). Statistical significance was determined using a unpaired two-tailed Student’s *t*-test. ****p < 0.01.


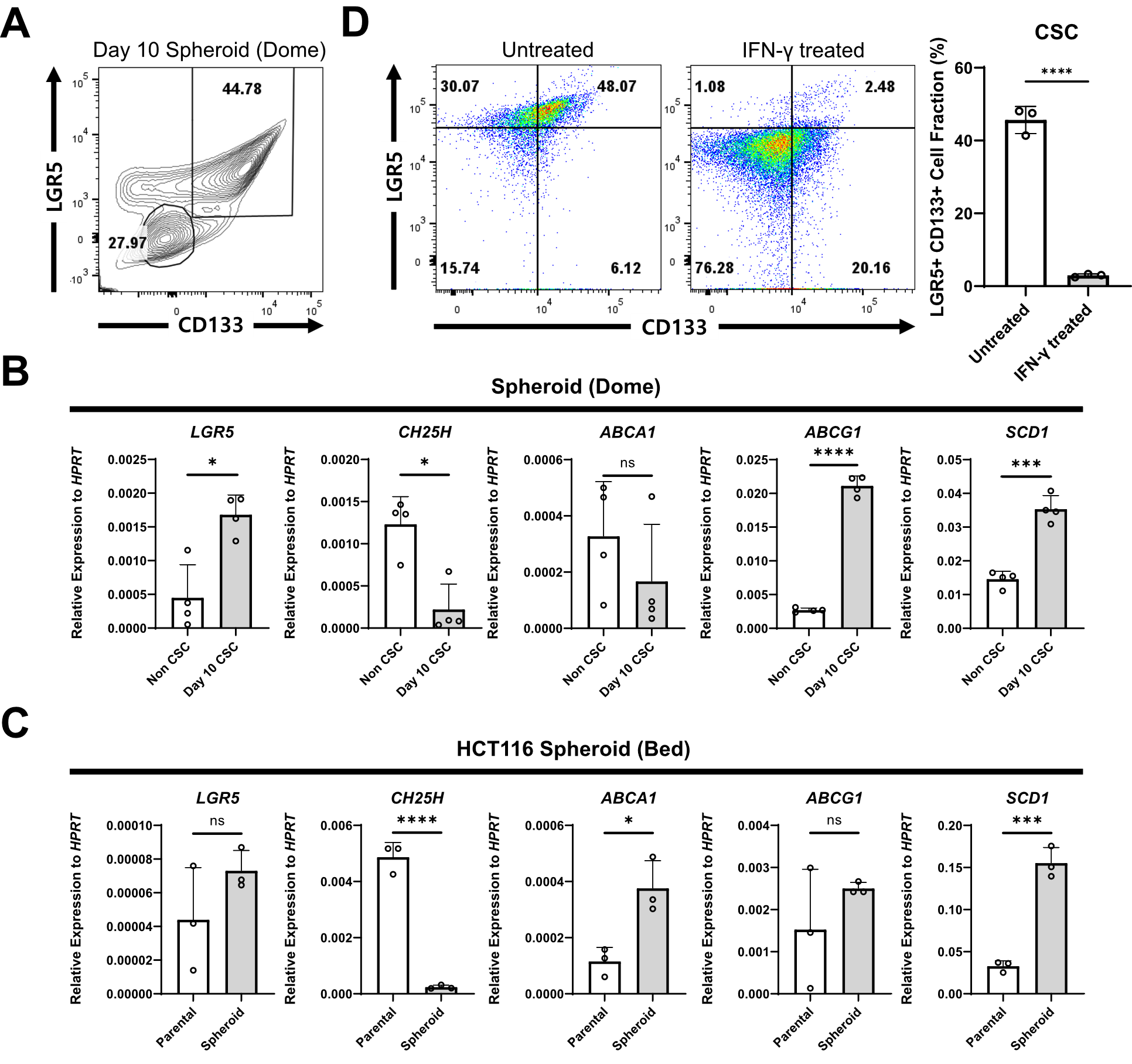


**Supplementary Figure 5. Analysis of lipid metabolic gene expression in colon cancer spheroids and IFN-γ-mediated reduction of CSC populations.**

**(A)** Representative flow cytometry plot illustrating the sorting strategy used to isolate CSC and non-CSC populations from day 10 spheroids based on CD133 and LGR5 expression, analogous to the gating strategy used for day 4 spheroids in Fig. 4A. **(B, C)** Quantitative RT–PCR analysis of the stem cell marker gene *LGR5* and lipid metabolism–related genes *(CH25H, ABCA1, ABCG1*, and *SCD1*) in CSC and non-CSC populations isolated from day 10 Caco-2 spheroids (B), and in HCT116 parental cells versus day 6 HCT116 spheroids (C). **(D)** Representative flow cytometry data showing CD133+LGR5+ CSC populations in cultured Caco-2 spheroids (dome) in the presence or absence of IFN-γ (1000 U/ml). Data are presented as mean ± SD. Statistical significance was determined using an unpaired two-tailed Student’s *t*-test. *p < 0.05, **p < 0.01, ***p < 0.001, ****p < 0.0001; ns, not significant.

**Supplementary Figure 6. Identification of CSCs in CRC patients single cell RNA-sequencing data**

**(A)** Global UMAP visualization of all cells, colored by cluster identity, respectively. **(B)** Feature plots and violin plots of canonical lineage markers across global clusters, including epithelial markers (*EPCAM*, *KRT8*, *KRT18*), immune marker (*PTPRC*), fibroblast marker (*COL1A1*), and endothelial marker (*PECAM1*), confirming accurate cell-type annotation. **(C)** Tumor epithelial cells were subsetted and re-clustered following batch correction using Harmony. CytoTRACE2 analysis revealed heterogeneous developmental potential. **(D)** Cells with high stemness scores were annotated as cancer stem-like cells (CSCs), supported by enriched expression of intestinal stem cell markers (*LGR5*, *PROM1*, *CD44*).

**Supplementary Figure 7. CH25H expression correlates with STAT1 signaling in human colorectal cancer.**

Continued analysis of scRNA-seq data in figure 5 and supplementary figure 5. **(A)** UMAP visualization of CSC and non-CSC populations within tumor epithelial cells. **(B)** Violin plots show increased WNT signaling activity, reduced STAT1 target gene activity, and decreased *CH25H* expression in CSCs compared to non-CSCs. **(C)** CAF populations were subsetted and were stratified into *CH25H*-high and *CH25H*-low groups. **(D)** Violin plots show that *CH25H*-high CAFs exhibit increased STAT1 expression and STAT1 target gene activity, consistent with observations in tumor epithelial cells. Statistical significance for comparisons of module scores between groups was assessed using the two-sided Wilcoxon rank-sum test. ****p < 0.0001; ns, not significant.
